# The no-touch vein graft harvesting technique for coronary artery bypass grafting surgery reduces mortality: a long-term follow-up study

**DOI:** 10.1186/1749-8090-8-S1-O177

**Published:** 2013-09-11

**Authors:** M Dreifaldt, L Bodin, D Ramos de Souza

**Affiliations:** 1 Cardio-Vascular Surgery, University Hospital, Orebro, Sweden; 2 Institute of Environmental Medicine, Unit of Intervention and Implementation Research, Karolinska Institute, Stockholm, Sweden

## Background

The saphenous vein is still the most widely used conduit in CABG surgery despite its poor long term patency. In the 1990's a new No-touch technique for saphenous vein graft harvesting for CABG was introduced where the vein graft is harvested with a pedicle of surrounding tissue. With the No-touch technique the vein graft does not go into spasm and the vessel wall does not have to be exposed to distension-induced trauma. A previous randomized trial has shown a significantly higher patency rate for vein grafts harvested with the No-touch technique compared to vein grafts harvested with conventional technique and the patency for No-touch vein grafts was comparable to the patency of the left internal thoracic artery, 8.5 years after surgery. The aim of this study was to evaluate whether the increased patency rate for No-touch vein grafts is reflected in clinical outcome.

## Methods

All patients (n=318) operated with CABG by the same surgeon and received either No-touch or conventional saphenous vein grafts, mainly as complementary grafting, between January 1, 1992, and December 31 1996 were included in the study. The patients were identified by 2 August 2007 through the Swedish Council registers regarding death and causes of death. The analysis used the Cox Proportional Hazard regression model to compare the two surgical techniques.

## Results

The adjusted hazard ratio for all-cause mortality for No-touch compared to conventional vein graft harvesting was 0.60 (0.36 – 0.98, 95 % CI) and for death in myocardial infarction the adjusted hazard ration for No-touch vein grafts was 0.36 (0.14 – 0.94, 95 % CI).

## Conclusions

This study shows that saphenous vein grafts, used for complementary grafting, harvested with No-touch technique, reduces all-cause mortality and death in myocardial infarction after CABG.

